# Two-Photon Absorption Activity of BOPHY Derivatives: Insights from Theory

**DOI:** 10.1021/acs.jpca.1c00756

**Published:** 2021-03-23

**Authors:** Elizaveta F. Petrusevich, Borys Ośmiałowski, Robert Zaleśny, Md. Mehboob Alam

**Affiliations:** † Theoretical Photochemistry and Photophysics Group, Faculty of Chemistry, Wrocław University of Science and Technology, Wyb. Wyspiańskiego 27, Wrocław PL−50370, Poland; ‡ Faculty of Chemistry, Nicolaus Copernicus University, Gagarina 7, Toruń PL-87100, Poland; ¶ Department of Chemistry, Indian Institute of Technology Bhilai, GEC Campus, Sejbahar, Raipur, Chhattisgarh 492015, India

## Abstract

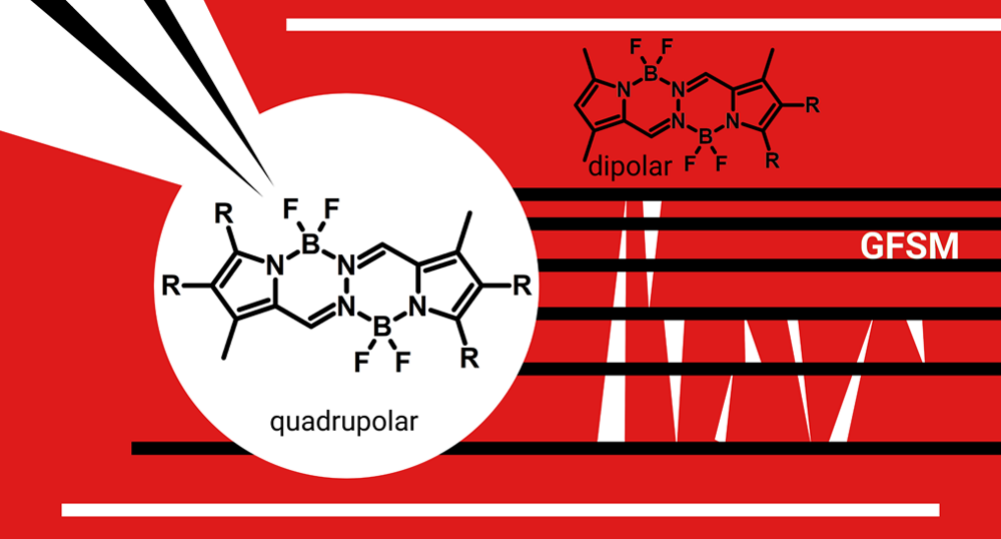

We present a theoretical study of a two-photon absorption (2PA) process in dipolar and quadrupolar systems containing two BF_2_ units. For this purpose, we considered 13 systems studied by Ponce-Vargas et al. [J. Phys. Chem. B2017, 121, 10850−108582913638310.1021/acs.jpcb.7b09698] and performed linear and quadratic response theory calculations based on the RI-CC2 method to obtain the 2PA parameters. Furthermore, using the recently developed generalized few-state model, we provided an in-depth view of the changes in 2PA properties in the molecules considered. Our results clearly indicate that suitable electron-donating group substitution to the core BF_2_ units results in a large red-shift of the two-photon absorption wavelength, thereby entering into the desired biological window. Furthermore, the corresponding 2PA strength also increases significantly (up to 30-fold). This makes the substituted systems a potential candidate for biological imaging.

## 1 Introduction

Materials showing large two-photon absorption (2PA) demonstrate a high potential for bioimaging applications due to several advantages that 2PA fluorescent probes have over one-photon absorption (1PA) probes.^1−3^ In particular, 2PA-based bioimaging provides a larger penetration depth of the excitation light into the tissue and shows higher spatial resolution due to the transparency of biological materials in the bioimaging spectral window.^4^ The use of infrared light leads to reduced photobleaching and photodamage of the biological samples. Taken together, these advantages make 2PA suitable for intrinsic 3D spatial resolution bioimaging. To that end, 2PA probes should have a large two-photon action cross section (product of two-photon absorption cross section and fluorescence quantum yield) exceeding 50 GM^5^ as well as an excitation wavelength within a window of 650–1100 nm.^6,7^ Photostability, noncytotoxicity, and water solubility are also required prerequisites for good 2PA probes.^8^

Difluoroborates as 2PA probes have a high potential in bioimaging applications, provided they meet the above-mentioned requirements.^9−13^ The main advantages of BF_2_-carrying molecules are high fluorescence quantum yield, strong absorbance and narrow emission bandwidth in the near-infrared region, high stability in the biological environment, and low toxicity. Difluoborates are small neutral compounds that can penetrate cell membranes. New derivatives with adjusted photophysical properties might be obtained by an easy functionalization. The simplest method to functionalize those molecules is the inclusion of the strong electron donating substituent and extension of the π-conjugation path.^14,15^ From the fundamental point of view, the 2PA cross section is proportional to the imaginary part of cubic hyperpolarizability;^16^ therefore, the design strategies of 2PA probes are based on combining electron donating and electron accepting groups, leading to a dipolar, quadrupolar, or octupolar skeleton of chromophores.^5,17−19^ The improvement of photophysical properties for bioimaging applications is possible by a variation of the substituents in the side chains. However, one should also not overlook the effect of the environment.

The efficient 2PA dyes exhibit significant charge transfer.^20^ To obtain that, strong electron donating substituents are used. However, in bioimaging applications, the water environment in the cells can cause hydrogen bonding to acceptor and donor substituents and aggregation of dyes, which will lead to a reduction of the 2PA cross section.^21^ It has been demonstrated that weaker donor substituents may not interact with solvents effectively in the excited state; thus, it helps to avoid the deterioration of 2PA properties.^22^ The solubility of dyes can be tuned by the attachment of long polyethylene glycol chains (or the cationic/anionic groups^23,24^), influencing the interaction by hydrogen bonding of those parts of the molecule that are not involved in the chromophore, and enhance biocompatibility.^8,25^ There are many works showing absorption near the first biological window with a high two-photon action cross section for a variety of donor–acceptor–donor (D–A–D) structures carrying a BF_2_ group as an acceptor. This is particularly true for 4,4-difluoro-4-bora-3a,4a-diaza-s-indacene (BODIPY) derivatives and difluoroboron β-diketonate dyes.^26−28^ There are successful studies on using different boron difluoride derivatives in bioimaging. BODIPY modified with phenylethynyl groups as the donor groups and two polyethylene glycol chains to afford water solubility showed good photostability in the cells, acceptable cytotoxicity, and a large 2PA cross section (500 GM) at 940 nm.^29,30^ Lipophilic pseudo di-BODIPY-based derivatives showed a huge two-photon action cross section (up to 870 GM) and effective diffusion into lipid rich organs^31^ although the higher value of the cross section may be attributed to the limited water presence in the lipids. Therefore, on the basis of the given characteristics of boron difluorides, organic dyes with a boron difluoride fragment inside the long conjugated π-system with terminal donor groups are perspective 2PA probes for bioimaging.

Among the plethora of difluoroborates, bis(difluoroboron)1,2-bis((1*H*-pyrrol-2-yl) methylene)hydrazine (BOPHY, a molecule with a D–A–A–D pattern), first reported in 2014,^32^ is of great interest to researchers due to its excellent photophysical properties. The design of new BOPHY derivatives is a promising route to optimize their properties for bioimaging applications. Recent theoretical work on substituted BOPHY derivatives showed a dependence of photophysical properties on the choice of the substituent.^33^ The study in question employed TD-DFT-SOS-CIS(D) analysis of 0–0 energies, a simulation of the vibrationally resolved spectra, a study of the electronic density difference, and charge transfer processes upon excitation.^33^ The present study is a significant extension of the preceding theoretical work, and it aims to assess the BOPHY derivatives with an eye toward their two-photon transition strengths. The set of studied compounds encompasses both symmetrical and asymmetrical π-conjugated BOPHY derivatives substituted with a wide range of donor groups in different positions. Some of the substituents were found to be beneficial in the case of the difluoroborate dyes as they showed excellent properties for bioimaging.^29,34,35^

Nowadays, electronic structure theories can be efficiently used for reliable predictions of various excited-state properties of molecules including two-photon transition strengths. In particular, thanks to developments by several research groups, it is now possible to determine the property in question at the post-Hartree–Fock level^36−39^ as well as within the density functional theory framework.^40−42^ In order to establish the structure-2PA relationships for π-conjugated BOPHY derivatives, we make use of these theoretical developments and employ the state-of-the-art implementation of the coupled-cluster CC2 model^37^ for the determination of 2PA strengths and we pinpoint the system-dependent 2PA properties in terms of electronic structure parameters using the recently developed generalized few-state model (GFSM).^43^

## 2 Computational Details

**Scheme 1:**
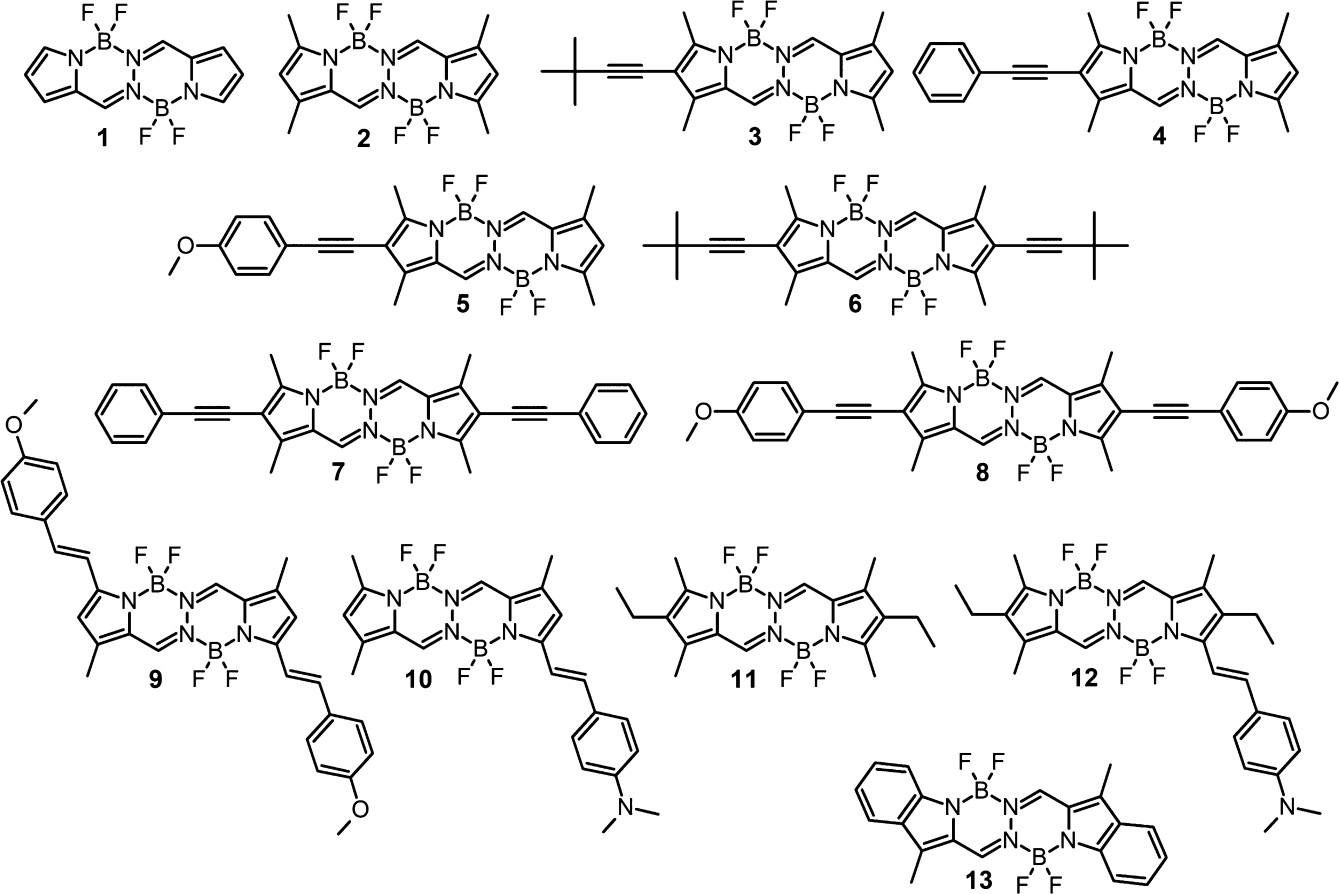
BOPHY Derivatives Studied in the Present Work

The structures of all 13 compounds, shown in Scheme 1, were optimized in the gas phase using the B3LYP functional^44^ and the 6-31G(d) basis set as implemented in the Gaussian 16 program.^45^ The stationary points obtained were confirmed to be minima by the evaluation of the Hessian. Gas-phase electronic structure calculations were performed at the optimized geometries to determine one- and two-photon absorption spectra. To that end, we used the RI-CC2 method as implemented in the TURBOMOLE program.^37,46^ In these calculations, the cc-pVDZ basis set^47^ and the corresponding recommended auxiliary basis set^48^ were used to determine the electronic structure. It follows from our earlier study on 2PA of organoboron complexes^43^ that the differences in two-photon transition strengths calculated at RI-CC2/cc-pVDZ and RI-CC2/aug-cc-pVDZ levels did not exceed 5%.

The coupled-cluster theory framework was used to determine the rotationally averaged two-photon transition strength (between states 0 and J) assuming one source of the linearly polarized monochromatic light beam:^36,37^
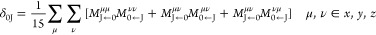
1where  and  stand for the left and right second-order transition moments, respectively. For one source of photons, i.e., , they read
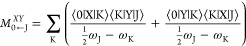
2
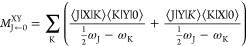
3

Inserting eqs 2 and 3 into eq 1, one can derive the expression for a generalized few-state model for non-Hermitian theories, where the left and right transition moments are different. The corresponding expression for the two-photon absorption strength is given by^43^
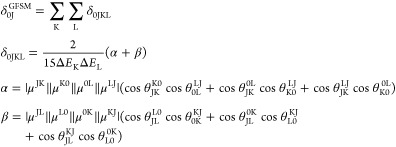
4

In the above expression, the superscripts distinguish between left (L0) and right moments (0L) and . The term  in eq 4 represents the angle between the transition dipole moment vectors μ^PQ^ and μ^RS^. In the case of theories with a Hermitian structure, i.e., where the left and right moments are equal, the above expression reduces to the one derived in a previous work^49^
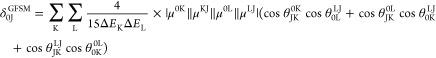
5Any number of intermediate states K and L can be chosen in the generalized few-state model expressions in eqs 4 and 5. In this work, we will make use of a two-state model (2SM) and a three-state model (3SM). In 2SM, K and L can be either the ground state 0 or the final excited state J, whereas in 3SM, an additional state is also included for both K and L. For instance, four terms contribute to the 2SM expression for δ(2SM): δ_0J00_, δ_0J0J_, δ_0JJ0_, and δ_0JJJ_ Similarly, in 3SM with intermediate state I, nine terms contribute to δ(3SM): δ_0J00_, δ_0J0I_, δ_0J0J_, δ_0JI0_, δ_0JII_, δ_0JIJ_, δ_0JJ0_, δ_0JJI_, and δ_0JJJ_.

## 3 Results and Discussion

The set of molecules selected for this study, also investigated by Ponce-Vargas et al.,^33^ allows us to establish the dependence of 2PA properties on the molecular architecture of the BOPHY derivatives. In what follows, we will concisely present the rationale behind the choice of this series. Compounds **1** (unsubstituted BOPHY) and **2** (1,3,6,8-tetramethyl BOPHY) are used in the current work as a starting point for other derivatives, created by adding electron-donating substituents. Molecules **3**–**5** and **6**–**8** are, respectively, 2-monosubstituted and 2,7-bisubstituted derivatives of tetramethyl BOPHY **2**; thus, on the basis of these two subsets of compounds, one can study the difference of two-photon transition strength between quadrupolar and dipolar structures. Moreover, an increase in the donor properties of the substituents in series **3**–**5** and **6**–**8** (*t*-butylethynyl, phenylethynyl, and *p*-methoxy-phenylethynyl, respectively) is expected to affect charge transfer upon excitations to higher electronic states and thereby nonlinear absorption spectra. Compounds **9** and **10** allow one to compare the quadrupolar structure (with two weak donor substituents) with the dipolar one (containing a substituent with strong electron-donating properties). The influence of steric effects due to substituents on the 2PA properties can be assessed on the basis of the example of the derivatives carrying an ethyl group and donor substituent in the BOPHY derivative **12** in comparison with structure **10** and core structure **11**. Structure **13** is a benzoannulated derivative of structure **11**. The said change should lead to the red-shift of absorption and an increase in chemical stability. Structure **13** is interesting for understanding the impact of central acceptor group modification on 2PA properties.

One-photon transition energies corresponding to the S_0_ → S_1_ electronic excitation are in the range of 2.62–3.28 eV, while for the S_0_ → S_2_ transition, they are in the range of 2.94–4.16 eV (see Table 1). The wavelengths corresponding to two-photon absorption lay in the first biological window, thus making some of the compounds good candidates for two-photon microscopy. Let us now discuss the dependence of the observed absorption energies on the structural features of the studied molecules. Reference structure **1** shows the largest value of excitation energy among the considered set, and it holds both for S_0_ → S_1_ and S_0_ → S_2_ transitions. Its methylated derivative (**2**) has a slightly lower value (roughly by 0.2 eV for S_0_ → S_1_ and less than 0.05 eV for S_0_ → S_2_ transitions). The addition of ethyl substituents (structure **11**) further slightly reduces the excitation energies. One finds benzannulation of structure **1** or **2** quite effective in lowering the energy gaps for both discussed transitions, which yields compound **13**. Note that the S_0_ → S_2_ excitation energy for quadrupolar molecule **13** has the lowest value among the considered set. When passing from **3** to **5** and from **6** to **8**, the electron donating properties of the substituents increase. Hence, it comes as no surprise that there is a decrease of the transition energy corresponding to S_0_ → S_1_ and S_0_ → S_2_ excitations. It is worth mentioning that, considering the substituent effect, the methyl groups present in the *t*Bu moiety can, in general, be treated as the electron-donating substituents. However, in the current structures, the effect is only local, opposite to the phenyl in which the influence is delocalized by conjugation.^50^ Nevertheless, the presence of the *t*Bu moiety can still be beneficial for an aggregation or solubility in nonpolar environments. Moreover, comparing each member from the series **3**–**5** (dipolar structures) with their counterparts in the series **6**–**8** (quadrupolar structures), one concludes that the latter molecules show lower energies than the dipolar structures. A further decrease in S_0_ → S_1_ and S_0_ → S_2_ can be achieved when passing from **8** to **9** with a different placement of the substituent. Note a slightly different linker as well. The use of the triple vs double carbon–carbon bond may be worth the synthetic effort in case one needs to avoid *trans*/*cis* photoisomerization. The comparison of quadrupolar structure **9** (containing weak electron-donating OMe substituents, σ = −0.27^51^) with dipolar structure **10** (containing strong electron-donating NMe_2_ substituents, σ = −0.83^51^) demonstrates that the excitation energy is lower in the case of the former (it holds for both electronic excited states). The addition of ethyl substituents to compound **10**, which gives **12**, leads to further, albeit insignificant, lowering of the S_0_ → S_2_ excitation energy (by 0.09 eV) and rising of the S_0_ → S_1_ excitation energy (by 0.02 eV). To sum up, on the basis of the BOPHY core, many structures with a wide range of excitation energies near the first biological window can be developed to be suitable for bioimaging applications. The lowest excitation energies are found for quadrupolar structures **9** (S_0_ → S_1_) and **13** (S_0_ → S_2_).

**Table 1:** One-Photon Excitation Energy (Δ*E*, eV), Oscillator Strength (*f*), and Average Two-Photon Transition Strength (δ × 10^–4^, au)^a^

	S_0_ → S_1_				S_0_ → S_2_			
	Δ*E*	*f*	δ	δ (2SM)	Δ*E*	*f*	δ	δ (3SM)
**1**	3.28	0.99	0.00		4.16	0.00	1.36	
**2**	3.09	1.07	0.00		4.13	0.00	1.70	2.25
**3**	3.00	1.24	0.12		3.74	0.05	2.78	3.71
**4**	2.96	1.38	0.37		3.64	0.07	4.71	6.64
**5**	2.92	1.37	1.02		3.54	0.12	6.49	8.63
**6**	2.92	1.43	0.00		3.57	0.00	5.78	9.44
**7**	2.84	1.74	0.00		3.40	0.00	11.93	21.34
**8**	2.79	1.79	0.00		3.28	0.00	18.39	32.99
**9**	2.62	1.97	0.00		3.20	0.00	40.94	43.90
**10**	2.70	1.29	9.44	12.90	3.27	0.47	12.20	13.94
**11**	3.04	1.20	0.00		3.88	0.00	2.02	
**12**	2.72	1.24	6.69	9.28	3.18	0.51	9.16	
**13**	2.78	1.19	0.00		2.94	0.00	0.90	

a Shown are also δ values obtained using the two-state model (2SM) and three-state model (3SM). See the text for more details.

**Figure 1:**
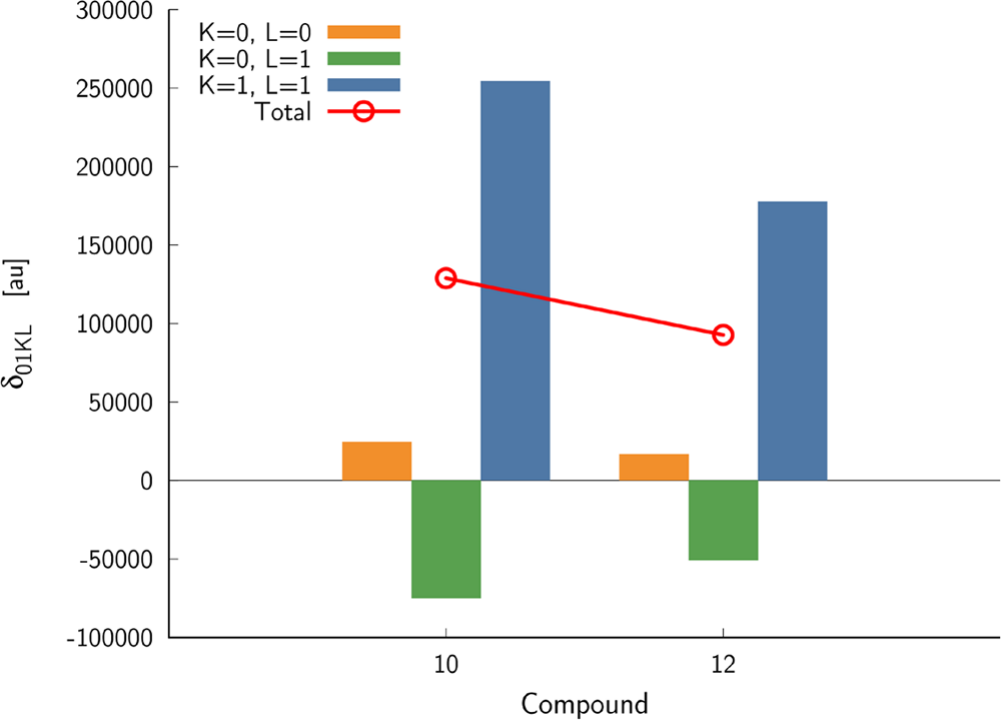
Summary of two-state model calculations corresponding to the two-photon S_0_ → S_1_ transition.

**Figure 2:**
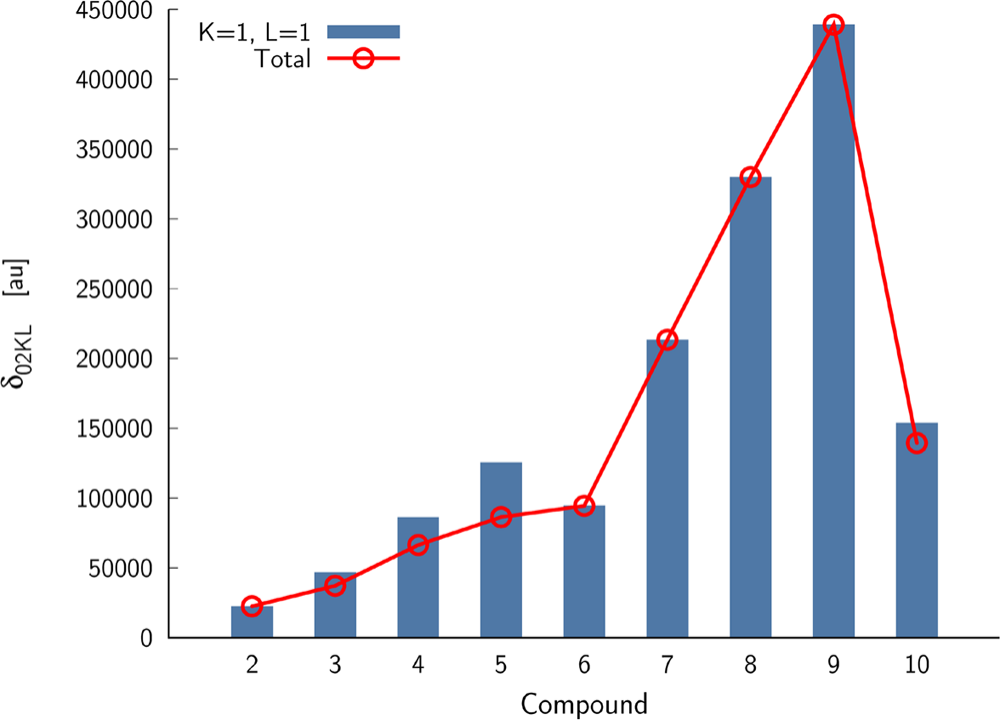
Summary of three-state model calculations corresponding to the two-photon S_0_ → S_2_ transition.

The summary of calculations of two-photon transition strengths is given in Table 1. In the case of the two-photon S_0_ → S_1_ transition, only dipolar structures (**3**–**5**, **10**, and **12**) exhibit nonzero strengths. The largest two-photon S_0_ → S_1_ absorption strengths are found for the latter two molecules carrying the strongest donating substituent (NMe_2_). From the structural point of view, **12** has bulky substituents, so the addition of two ethyl groups to **10** causes steric effects (twisting around single bonds by ca. 17° and 6° for core —CH= and =CH—C_6_H_4_ single bonds, respectively). The effect of the twist may be used in bioimaging as a tool to change the properties of the fluorophore, making it responsive due to the PICT (planar intramolecular charge transfer) mechanism.^52^ It is also worth recalling that alkyl groups (Et in this case) are electron donating substituents. Thus, the overall electron accepting properties of the BF_2_ core are decreased and so is the effectiveness of the charge transfer. The latter effect is expected to contribute to a larger extent to the observed decrease of two-photon transition strength, in comparison with geometry distortion, when passing from **10** to **12**. For dipolar structures **10** and **12** with meaningful two-photon S_0_ → S_1_ transition strengths, we also performed calculations on the basis of the generalized few-state model. It comes as no surprise that the two-state model (2SM), including the ground and final electronic state, is sufficient to reliably predict the property in question. Figure 1 shows the summary of 2SM-based calculations of the two-photon S_0_ → S_1_ transition for the two dipolar structures **10** and **12**. As seen, the dominant term contributing to the two-photon transition strength is δ_0111_, which according to eq 4, has a product of |μ^01^|^2^ (square of the transition dipole moment between S_0_ and S_1_) and |μ^11^|^2^ (square of the dipole moment in the S_1_ excited state). In the case of compound **10**, the value of the δ_0111_ term is much larger than that for compound **12**, and this is due to the contribution from |μ^11^|^2^. We noticed that the cross term δ_0101_ in both **10** and **12** contributes destructively; i.e., the value is negative. However, relatively in **12**, the decrease due to δ_0101_ is larger than that in **10**. Table 1 also contains the values of two-photon S_0_ → S_2_ transition strengths. All dipolar structures, but **10**, exhibit transition strengths smaller than 10^5^ au. In the case of **10**, one finds comparable values of δ for both considered electronic transitions. In the case of quadrupolar structures, the considered chemical modifications induce huge variations in S_0_ → S_2_ two-photon transition strengths, e.g., from 1.36 × 10^4^ au (**1**) up to 40.94 × 10^4^ au (**9**). To shed more light on these substituent-induced changes, we performed three-state model analysis. To that end, we considered several intermediate states choosing the one that yields the δ value closest to the response theory value. In fact, for all 3SM data shown in Table 1, the S_1_ electronic excited state was chosen as the intermediate state. Figure 2 shows the summary of the 3SM calculations. In particular, it contains the key contribution to the two-photon S_0_ → S_2_ transition strength reported in Table 1, which is δ_0211_. This term is a product of |μ^01^|^2^ and |μ^12^|^2^. For dipolar structures (**3**–**5** and **10**), the δ_0211_ term is larger than the corresponding total 3SM value. In the case of quadrupolar structures (**2**, **6**–**9**), it is the only contributing term due to the symmetry. As we clearly see, when passing from compound **7** to **9**, this term significantly increases, paralleling the trend predicted by the response theory accounting for the full spectrum of Hamiltonian.

## 4 Summary and Conclusions

In conclusion, we have studied the one- and two-photon absorption processes in the BF_2_ unit containing dipolar and quadrupolar molecules using the state-of-the-art RI-CC2 method. Our results indicate that the energy gap between the ground state and the two-photon active state in the stated molecules can be controlled in various ways including the benzoannulation process and by placing the electron donating group at different positions. The two-photon activity in such compounds can be affected due to various substitutions as well as by varying the steric effect. A decrease in the steric crowd leads to more planar geometry, which in turn may increase the two-photon activity. We have observed that through proper substitution of an electron-donating group in the core bis-BF_2_ containing unit it is possible to red-shift the two-photon absorption wavelength from 596 to 775 nm so that it is located in the desired biological window. We have further observed that the first two most two-photon active compounds contain an ether linkage. This linkage can be extended experimentally leading to a highly two-photon active molecule being soluble in water. An important finding of this work is that the presented structural modifications can boost the two-photon transition strength even by a factor of 30.

## Supporting Information Available

Components of two-state model δ_0JKL_ terms contributing to the two-photon activity in molecules **10** and **12**, components of three-state model δ_0JKL_ terms contributing to the two-photon activity in the remaining molecules, and contributing one-photon left- and right-transition moments (PDF)
