# Supplementary material for: Two-Photon Absorption Activity of BOPHY Derivatives: Insights from Theory
Source: J Phys Chem A. 2021 Mar 23;125(12):2581–7. doi: 10.1021/acs.jpca.1c00756 (PMC8154621; doi:10.1021/acs.jpca.1c00756)
Supplement: Supplementary file 1 — jp1c00756_si_001.pdf [file jp1c00756_si_001.pdf]

Supporting Information: Two-Photon Absorption Activity of BOPHY Derivatives: Insights from Theory

Elizaveta F. Petrusevich,[†] Borys Ośmiałowski,[‡] Robert Zaleśny,[*,†] and Md. Mehboob Alam[*,¶]

[†]*Theoretical Photochemistry and Photophysics Group, Faculty of Chemistry, Wrocław University of Science and Technology, Wyb. Wyspiańskiego 27, PL–50370 Wrocław, Poland*

[‡]*Faculty of Chemistry, Nicolaus Copernicus University, Gagarina 7, PL-87100 Toruń, Poland*

[¶]*Department of Chemistry, Indian Institute of Technology Bhilai, GEC Campus, Sejbahar, Raipur, Chhattisgarh, India – 492015*

E-mail: robert.zalesny@pwr.edu.pl; mehboob@iitbhilai.ac.in

Table S1: Two-photon transition strengths ($\delta_{0JKL}\times10^{-4}$, au) contributing to δ(2SM) corresponding to the $S_0 \rightarrow S_1$ transition for the structures **10** and **12**.

	**10**	**12**
δ_{0100}	2.45	1.68
δ_{0101}	-7.50	-5.08
δ_{0110}	-7.50	-5.08
δ_{0111}	25.45	17.76
δ(2SM)	12.90	9.28

Table S2: Two-photon transition strengths ($\delta_{0JKL}\times10^{-4}$, au) contributing to the δ(3SM) corresponding to the $S_0 \rightarrow S_2$ transition for the structures **2-10**.

	**2**	**3**	**4**	**5**	**6**	**7**	**8**	**9**	**10**
δ_{0200}	0.00	0.00	0.00	0.01	0.00	0.00	0.00	0.00	0.27
δ_{0220}	0.00	0.00	0.01	-0.07	0.00	0.00	0.00	0.00	-0.38
δ_{0210}	0.00	0.02	-0.05	0.28	0.00	0.00	0.00	0.00	0.39
δ_{0202}	0.00	0.00	0.01	-0.07	0.00	0.00	0.00	0.00	-0.38
δ_{0222}	0.00	0.07	0.13	0.51	0.00	0.00	0.00	0.00	0.63
δ_{0212}	0.00	-0.53	-1.02	-2.43	0.00	0.00	0.00	0.00	-1.19
δ_{0201}	0.00	0.02	-0.05	0.28	0.00	0.00	0.00	0.00	0.39
δ_{0221}	0.00	-0.53	-1.02	-2.43	0.00	0.00	0.00	0.00	-1.19
δ_{0211}	2.25	4.66	8.62	12.55	9.44	21.34	32.99	43.90	15.37
δ(3SM)	2.25	3.71	6.64	8.63	9.44	21.34	32.99	43.90	13.94

Table S3: One-photon left and right transition moments ($|M_{IJ}|$, au) contributing to the terms listed in Tables 1 and 2.

IJ	**2**	**3**	**4**	**5**	**6**	**7**	**8**	**9**	**10**	**12**
00	0.00	0.11	0.15	0.67	0.00	0.00	0.00	0.00	2.43	2.24
01	2.94	3.18	3.37	3.38	3.45	3.85	3.91	4.24	3.36	3.28
02	0.00	0.59	0.67	0.91	0.00	0.00	0.00	0.00	1.89	
10	4.82	5.30	5.65	5.69	5.81	6.51	6.69	7.23	5.82	5.65
11	0.00	0.81	1.10	2.55	0.00	0.00	0.00	0.00	6.71	6.02
12	1.56	2.35	3.08	3.77	3.11	4.30	5.32	4.89	3.80	
20	0.00	0.97	1.11	1.50	0.00	0.00	0.00	0.00	3.10	
21	1.81	2.52	3.21	3.86	3.28	4.38	5.35	5.29	4.05	
22	0.00	2.82	3.22	4.44	0.00	0.00	0.00	0.00	3.81	
